# A conceptual exploration of generative AI-induced cognitive dissonance and its emergence in university-level academic writing

**DOI:** 10.3389/frai.2025.1573368

**Published:** 2025-06-17

**Authors:** Carl Errol Seran, Myles Joshua Toledo Tan, Hezerul Abdul Karim, Nouar AlDahoul

**Affiliations:** ^1^Biology Program, College of Arts and Sciences, University of St. La Salle, Bacolod City, Philippines; ^2^Department of Natural Sciences, College of Arts and Sciences, University of St. La Salle, Bacolod City, Philippines; ^3^Faculty of Education, University of the Philippines Open University, Los Baños, Philippines; ^4^Department of Electrical and Computer Engineering, Herbert Wertheim College of Engineering, University of Florida, Gainesville, FL, United States; ^5^Department of Epidemiology, College of Public Health and Health Professions and College of Medicine, University of Florida, Gainesville, FL, United States; ^6^Department of Chemical Engineering, College of Engineering and Technology, University of St. La Salle, Bacolod City, Philippines; ^7^Department of Electronics Engineering, College of Engineering and Technology, University of St. La Salle, Bacolod City, Philippines; ^8^Yo-Vivo Corporation, Bacolod City, Philippines; ^9^Centre for Image and Vision Computing, Centre of Excellence for Artificial Intelligence, Faculty of Artificial Intelligence and Engineering, Multimedia University, Cyberjaya, Malaysia; ^10^Department of Computer Science, Division of Science, New York University Abu Dhabi, Abu Dhabi, United Arab Emirates

**Keywords:** academic integrity, AI literacy, cognitive dissonance, ethical AI use, generative AI (GenAI), learning behavior, university writing

## Introduction

University-level academic writing is a form of scholarly communication that demands precision, clarity, and adherence to established conventions. It is a skill fundamental to science education, best honed through continuous practice (Moskovitz and Kellogg, 2011). However, rising academic demands and evolving educational environments, such as shifts to online learning, introduce new challenges. Students often view writing as a daunting task, largely due to insufficient instruction bridging technical composition and creative expression in their coursework (Stride, 2024). Non-native speakers find this struggle compounded, grappling with the linguistic precision and stylistic conventions of academic writing (Nazaroff, 2011). Furthermore, traditional academic writing has also suffered from an overemphasis on technical correctness at the expense of fostering creative expression. In many cases, the perceived marginal role of writing in research and teaching has led educators to delegate writing instruction solely to language departments (Alley, 2024). Such delegation has resulted in a fragmented approach to teaching writing skills, leaving many students underprepared. In the advent of Generative Artificial Intelligence (GenAI), some students now assume that technology can fully compensate for their underdeveloped writing abilities, inadvertently undervaluing the importance of building a strong writing foundation.

GenAI tools—such as ChatGPT™ by OpenAI, Gemini™ by Google, and Claude™ by Anthropic—have emerged as practical aids for organizing and simplifying writing tasks such as article reviews, lab reports, and research papers (Essel et al., 2024). These technologies help reduce the cognitive load by shifting focus from the mechanical aspects of writing to higher-level critical analysis and interpretation (Olatunbosun and Nwankwo, 2024). However, their integration into academic practice often creates an ambiguous boundary between human intellectual effort and machine assistance (Amoozadeh et al., 2024), hence leading to what we term as cognitive dissonance (CD), a classic social psychology theory introduced by Festinger in 1957. CD is the inconsistency in thoughts, actions, or behavior that leads to a tension or discomfort from holding contradictory beliefs (Hilberg, 2017). This tension is resolved by changing our thoughts or behaviors, adding a new thought, or rationalizing the inconsistencies (Oxoby and Smith, 2014).

To date, no consensus has been reached regarding the manifestation of cognitive dissonance in the integration of GenAI in university-level academic writing. Here, we provide a novel perspective on how CD emerges with the integration of GenAI in university-level academic writing. We posit that GenAI both triggers and exacerbates pre-existing tensions, reshaping academic writing dynamics. Furthermore, exploring pedagogical frameworks like constructivism may offer pathways to mitigate these emerging challenges and help students integrate GenAI tools responsibly.

## Understanding cognitive dissonance in academic writing

Cognitive dissonance has long been a topic of concern in academic writing. Traditionally, it refers to the psychological tension experienced when students' ideals clash with the practical constraints they face (McGrath, 2017). Operationalized as a state of psychological discomfort (Vaidis and Bran, 2019), CD in academic writing often emerges from the conflicting demands between producing high-quality, original work and managing limited time, resources, or guidance. The magnitude of the dissonance experienced depends on the importance of the conflicting cognitions and the proportion of dissonant vs. consonant thoughts related to a specific behavior or belief (Morvan and O'Connor, 2017). When faced with this discomfort, individuals are strongly motivated to reduce it. Classic dissonance reduction strategies include: (1) changing one of the dissonant cognitions (e.g., altering one's attitude toward academic integrity), (2) changing behavior to align with cognitions (e.g., stopping the use of GenAI in ways perceived as dishonest), (3) adding new consonant cognitions to justify the behavior (e.g., emphasizing the time saved allows focus on higher-level thinking), or (4) trivializing the conflict itself (e.g., deciding that the specific assignment is not important anyway; McGrath, 2017; Stephens, 2017). Understanding these mechanisms is crucial for analyzing how students might react to the internal conflicts provoked by GenAI use in academic writing. For example, ethical dilemmas may arise when the pressure to publish meets the rigorous standards of originality and research integrity (Schrems and Upham, 2020). This internal conflict can lead students to adopt selective coping strategies, such as focusing only on information that confirms their existing beliefs (Metzger et al., 2020), or in some cases, resorting to dishonest practices (Stephens, 2017). In traditional academic settings, CD can serve as both a source of psychological stress and a catalyst for personal growth. This occurrence prompts students to recalibrate their beliefs and strive for higher standards. However, the introduction of GenAI into this landscape introduces new dimensions of dissonance that are still not fully understood.

## GenAI-induced cognitive dissonance: triggers and exacerbated tensions

### GenAI as a trigger for cognitive dissonance

A trigger, in this context, refers to any factor that disrupts an individual's core beliefs and values, creating psychological tension. GenAI's efficiency, while beneficial, directly conflicts with the academic values of originality, effort, and intellectual ownership. Empirical studies (Chan, 2025; Playfoot et al., 2024) provide evidence that GenAI use can lead to what some describe as “AIgiarism”—a nuanced form of plagiarism where students, while disapproving of overt AI-generated content, find themselves ambivalent about employing AI for paraphrasing or idea generation. For instance, Chan (2025) found significant ethical ambivalence regarding AI use for idea generation vs. direct submission. Moreover, Playfoot et al. (2024) explored the adoption of ChatGPT^TM^ for writing tasks. The 467 participants expressed ambivalence toward ethical boundaries where 68% recognized GenAI use conflicted with academic integrity, while 52% prioritized convenience and perceived low detection risks. This exemplifies CD as the efficiency of GenAI contradicts with values like originality and effort.

This ethical ambivalence and conflict between values and convenience exemplify CD in action: the psychological discomfort arises from incompatible cognitions (e.g., “Upholding academic integrity is important” vs. “Using GenAI for this task is efficient and tempting”). According to theory, this discomfort motivates individuals to reduce the dissonance, potentially by downplaying the importance of integrity, justifying the AI use, or altering their behavior (McGrath, 2017; Stephens, 2017).

### GenAI exacerbating pre-existing tensions

Beyond serving as an initial trigger, GenAI can also exacerbate pre-existing tensions in academic writing. While academic challenges such as balancing clarity with complexity have long existed, GenAI potentially heightens these issues. For instance, Zhai et al. (2024) highlighted a student preference for efficiency over deep cognitive engagement; leveraging GenAI for efficiency could correlate with this trend. Similarly, Ironsi and Solomon Ironsi (2025) observed a conflict between perceived GenAI benefits for clarity and concerns about independent skill development. Hutson (2024) added that habitual GenAI use encourages cognitive shortcuts, potentially undermining learning.

**Figure 1:**
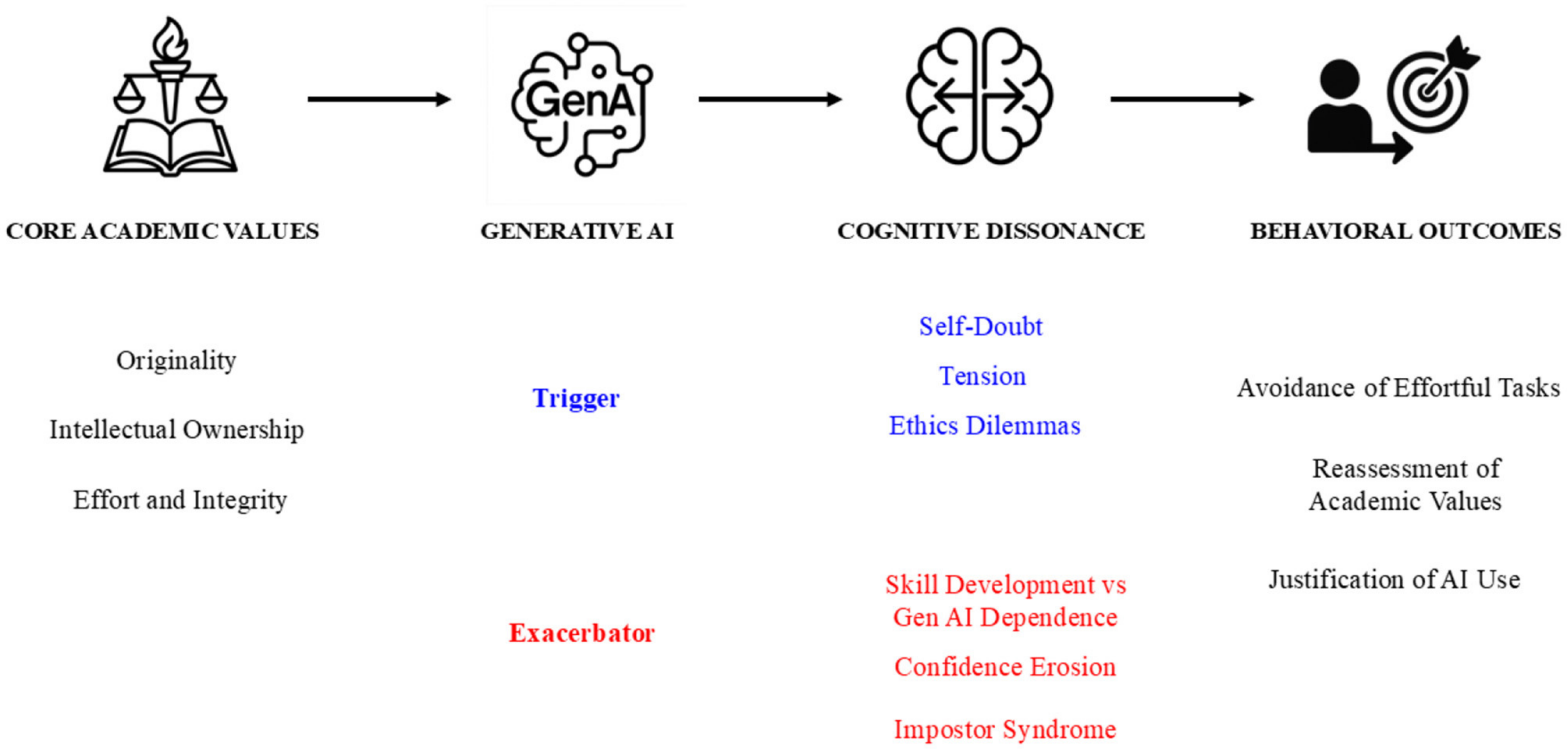
Hypothetical construct of GenAI-induced CD. GenAI is both a trigger and an exacerbator of CD in academic writing. The figure illustrates how the integration of GenAI disrupts core academic values, leading to CD. This is manifested as self-doubt, ethical dilemmas, and confidence erosion, among others. These psychological tensions influence behavioral outcomes, including avoidance of effortful tasks, reassessment of academic values, and justification of AI use. The process is further exacerbated by increasing dependence on GenAI, which exacerbates existing struggles related to skills development and academic integrity.

These findings suggest GenAI heightens dissonance related to self-efficacy and learning goals. The conflict between the desire for genuine skill development (“I need to learn to write independently”) and the reliance on an external tool (“GenAI helps me produce clear text easily”) creates discomfort. This may lead students to adopt dissonance-reducing strategies such as rationalizing their dependence on AI, minimizing the value of independent writing skills for certain tasks, or experiencing increased anxiety and self-doubt (related to impostor syndrome; McGrath, 2017; Stephens, 2017). In this light, GenAI is not merely introducing new ethical dilemmas but is also potentially exacerbating existing academic pressures and internal conflicts. The relationship between GenAI, CD and behavioral outcomes in academic settings is visually summarized in Figure 1, which illustrates GenAI as both a trigger and exacerbator of CD.

### Mitigating cognitive dissonance through constructivist pedagogy

Addressing the cognitive dissonance triggered or exacerbated by GenAI requires pedagogical approaches that go beyond simply regulating tool use. Constructivist pedagogy offers a promising framework because its core tenets directly counter some of the sources of dissonance. Constructivism emphasizes that learners actively construct their own understanding through experience, reflection, and social interaction, rather than passively receiving information. This contrasts sharply with the potential for passive reliance on GenAI outputs.

Specifically, constructivist approaches may mitigate GenAI-related CD in several ways:

1. *Emphasis on process and reflection:* by valuing the learning process, reflection, and metacognition (Alt et al., 2022), constructivism helps students focus on their own intellectual journey. This can reduce the dissonance arising from the conflict between using an 'easy' tool and the value placed on effort and genuine understanding. Reflecting on *how* and *why* they use GenAI allows students to integrate the tool into their process in a way that feels authentic and justifiable, reducing integrity-related dissonance.
2. Active engagement and authentic tasks: constructivist learning often involves complex, authentic tasks that require critical thinking, problem-solving, and synthesis—skills where current GenAI may be less effective as a complete substitute. Engaging deeply with such tasks reinforces the value of human intellect and skill development, potentially reducing dissonance related to fears of skill atrophy or over-reliance (Tan and Maravilla, 2024). Active construction of knowledge fosters a sense of ownership that can counteract feelings of inadequacy or impostor syndrome sometimes associated with heavy GenAI use.
3. *Fostering internal motivation:* constructivist environments aim to foster intrinsic motivation by making learning meaningful and relevant. When students are internally motivated to understand and create, the external pressure to simply complete tasks efficiently (a potential trigger for dissonance when using GenAI shortcuts) may be lessened.

Therefore, applying constructivist principles to assignment design and classroom practice could help students navigate the use of GenAI more ethically and productively, transforming potential dissonance into opportunities for meaningful learning about both the subject matter and the responsible use of technology.

## Call to action

Below are practical strategies, informed by dissonance reduction principles and constructivist pedagogy, to help navigate GenAI use in academic writing.

### Establish AI policies and literacy programs to curb over-reliance

Universities need clear policies on transparency, attribution, integrity, and acceptable levels of GenAI assistance (e.g., distinguishing support from unacceptable content generation) to mitigate ethical dilemmas. By reducing ambiguity about acceptable use and providing clear behavioral pathways aligned with academic values, such policies can decrease the internal conflict (CD) students experience when balancing the utility of GenAI with the demands of integrity.

Alongside these policies, professional development for faculty is essential. Training in prompt engineering, for instance, can enable instructors to design precise, thought-provoking prompts that stimulate critical thinking and may even require students to evaluate or critique GenAI outputs (Lee and Palmer, 2025). While student over-reliance on GenAI for task completion is a primary concern leading to dissonance, instructor use of prompt engineering serves a distinct pedagogical purpose. It focuses on leveraging AI as a design tool to enhance critical thinking challenges and model thoughtful, ethical engagement with the technology, rather than replacing the student's learning process or facilitating academic dishonesty. Integrating prompt-generation practices or using GenAI for formative feedback can reinforce the view of GenAI as a supportive tool rather than a substitute for original thought.

Dedicated AI literacy modules should teach critical evaluation of AI content, its limitations, and differentiation from human effort (Wang et al., 2024). For instance, students might compare ChatGPT-generated summaries against scholarly sources or analyze outputs for bias. Practical activities like peer review focusing on the ethical use of sources (including AI) and analysis of information quality can help students build confidence and make informed decisions about GenAI use, further reducing potential CD.

### Integrate reflective pedagogy to mitigate ethical dilemmas and preserve confidence

Instructors should incorporate reflective exercises related to students' writing processes. Reflective pedagogy boosts metacognitive awareness through self-assessment of GenAI's role (Alt et al., 2022). For example, after comparing GenAI outputs with other references, students might answer questions like, “Did GenAI's structure enhance or override my reasoning?” or “Did this process clarify my stance or obscure my effort?” Such reflection can reduce ethical tension and reinforce confidence in their own writing. Maintaining a digital journal comparing AI-assisted and manual drafts curbs over-dependence (Kim et al., 2025); peer review of excerpts can foster accountability. Instructors should participate in workshops featuring role-playing, reflective prompts, and journal analyses to better guide students in balancing AI use with personal insights.

### Redesign hybrid writing to reclaim critical thinking and uphold academic integrity

Educators must create a hybrid writing process that combats CD and skill degradation. One approach is to require students to submit two drafts: one incorporating AI assistance and a second, a manually refined version. For example, students could use GenAI to generate an initial literature review but manually craft the final version. This constructivist-based hybrid model helps students develop arguments from AI scaffolds (Tan and Maravilla, 2024). Updated rubrics should reward human effort, ensuring GenAI acts as support, not a substitute.

## Conclusion

The emergence of GenAI-induced CD in academic writing presents an urgent challenge for higher education. Universities must respond proactively by embedding strategies rooted in dissonance reduction and constructivist pedagogy. Explicitly establishing clear AI literacy programs, updated academic integrity policies, and reflective teaching practices will directly empower students and educators alike. These measures will further build their confidence and skills to responsibly use GenAI as a powerful tool for academic writing.
